# Functional Analyses of *RUNX3* and *CaMKIINα* in Ovarian Cancer Cell Lines Reveal Tumor-Suppressive Functions for *CaMKIINα* and Dichotomous Roles for *RUNX3* Transcript Variants

**DOI:** 10.3390/ijms19010253

**Published:** 2018-01-15

**Authors:** Karolin Heinze, Daniel Kritsch, Alexander S. Mosig, Matthias Dürst, Norman Häfner, Ingo B. Runnebaum

**Affiliations:** 1 Department of Gynecology, Jena University Hospital—Friedrich Schiller University Jena, 07747 Jena, Germany; karolin.heinze@med.uni-jena.de (K.H.); daniel.kritsch@uni-jena.de (D.K.); matthias.duerst@med.uni-jena.de (M.D.); 2 Department of Biochemistry II, Jena University Hospital—Friedrich Schiller University Jena, 07747 Jena, Germany; alexander.mosig@med.uni-jena.de

**Keywords:** epithelial ovarian cancer, platin resistance, *RUNX3*, *CaMKIINα*, tumor suppressor

## Abstract

(1) Background: Epithelial ovarian cancer (EOC) is the most lethal cancer of the female reproductive system. In an earlier study, we identified multiple genes as hypermethylated in tumors of patients with poor prognosis. The most promising combination of markers to predict a patient’s outcome was *CaMKIINα* and *RUNX3*. Aim of this study was to functionally validate the importance of both genes. (2) Methods: IC_50_ measurements, cell cycle distribution-, proliferation, and migration experiments were conducted after transgene overexpression in two EOC cell lines. (3) Results: We showed that *CaMKIINα* has tumor suppressive functions in vitro and reduces proliferation, migration, and colony formation. However, it had no effect on the reversion of the resistance to cisplatin. *RUNX3* exhibited dualistic functions related to cisplatin sensitivity and migration capacity, depending on the respective transcript variant (TV). A2780 cells expressing *RUNX3* TV2—the promoter of which harbors a CpG (5′-C-phosphate-G-3′) island and is potentially inactivated by hypermethylation—exhibited increased cisplatin sensitivity and reduced migration properties. However, *RUNX3* TV1, not affected by CpG island methylation could be characterized as mediating resistance and enhancing migration in A2780. The higher resistance of *RUNX3* TV1 transfected cells correlates with a reduction of cell proliferation. Moreover, *RUNX3* TV1 expressing cells exhibit a reduced cell cycle arrest at the gap-2 or mitosis phase (G2/M) under cisplatin treatment comparable to resistant A2780 subcultures. (4) Conclusion: It appears that *CaMKIINα* and *RUNX3* TV2 can reduce the malignant potential of EOC cells.

## 1. Introduction

In developed countries, ovarian cancer is the third most common gynecological malignancy [1,2]. About 95% are of epithelial origin and can be classified into distinct subtypes. Within these subtypes, the high-grade serous EOC represent the biggest fraction and are at the same time also the most problematic one [3]. Because approximately 60–80% of the EOC patients are diagnosed in late stages such as T3, the 5-year-survival-rate (5yr-SR) is lower than 40% [2,4,5]. Besides the lack of specific early symptoms and missing screening opportunities which contribute to late diagnosis, the frequent relapse with a chemo-resistant disease is also responsible for the high mortality rate [6]. While just 3.6% of all cancer incidences worldwide are due to ovarian cancer, it accounts for 4.3% of all cancer death—the highest mortality rate of gynecological cancers in women [7]. Over the last decades the overall 5yr-SR of EOC diagnosed women has not changed much since the introduction of platinum-based chemotherapeutics [8,9,10]. Importantly new treatment options i.e., anti-angiogenesis and PARP-inhibitor treatments may improve the 5yr-SR in the future. The current standard therapy is initial surgery to reduce the tumor burden macroscopically followed by adjuvant therapy with carboplatin or cisplatin in combination with other antineoplastic drugs, e.g., taxane, doxorubicin. After an initial response, a high number of patients relapse with chemotherapeutic-resistant disease within a median of 18 months [6]. Reliable biomarkers are needed to improve and adjust the patients’ therapy.

The hypothesis that aberrant DNA methylation might be useful to establish new biomarkers for EOC has been proposed from several research groups [11,12]. In a previous study we identified and validated five potential marker genes by scanning 48 primary EOC tumors. It could be demonstrated that the hypermethylation of *RUNX3* and *CaMKIINα* is a prognostic marker for EOC [13]. The marker combination *CaMKIINα* and *RUNX3* demonstrated a sensitivity of 40%, and a specificity of 100% to identify patients with progression-free survival of <3 years. Furthermore, when performing Kaplan–Meier analysis it could be shown that the unmethylated cohort had a significantly prolonged progression-free survival in contrast to the methylated one, independent from the clinical parameters such as the resection status [13]. CaMKIINα is one of two endogenous inhibitors of the Calmodulin-dependent protein kinase-II [14,15]. Widely, its expression in other tumor entities is described to have positive effects on the patients’ outcome, indicating a tumor suppressor function [16,17,18]. RUNX3 on the other hand, is a transcription factor prone to have a dualistic association in carcinogenesis [19]. Analyses of RUNX3 isoforms described different functions during immune cell development and gastric cancer susceptibility [20,21]. The mechanism and functional involvement of CaMKIINα and RUNX3 isoforms in ovarian carcinogenesis is not fully understood until now. Here we aimed to determine the functional contribution of both genes to cellular properties i.e., cell proliferation, migration and cisplatin response. Results revealed that *CaMKIINα* has tumor suppressive functions and reduces proliferation, migration and colony formation but does not restore the cisplatin sensitivity of resistant cells. *RUNX3* transcript variants exhibited dichotomous functions in regard to cisplatin sensitivity and migration of A2780 cells.

## 2. Results

Aim of the present study was the functional characterization of *CaMKIINα* and *RUNX3* in ovarian cancer cell lines—specifically in isogenic pairs of platinum-sensitive and -resistant A2780 and SKOV3. Both genes were identified as hypermethylated in EOC patients with poor prognosis implicating a potential contribution to aggressive tumor biology or chemotherapeutic resistance. Moreover, *CaMKIINα* hypermethylation was identified during resistance development in SKOV3 cells resulting in an inhibition of gene expression under cisplatin treatment [13]. Additionally, neither parental nor resistant A2780 cells do express *CaMKIINα*. Therefore, we hypothesized a potential contribution of *CaMKIINα* to cisplatin sensitivity. To investigate the role of *CaMKIINα*, the gene was overexpressed in the resistant cell cultures, allowing for a reversion of cisplatin resistance. In contrast to *CaMKIINα*, *RUNX3* methylation and expression levels were not altered during in vitro cisplatin resistance development in A2780 and SKOV3. Moreover, A2780 are negative for *RUNX3*, and SKOV3 express low levels of this gene [22]*,* resembling EOC with *RUNX3* loss. Thus, we aimed to analyze consequences of *RUNX3* overexpression in parental A2780 and SKOV3 cells to investigate the ability of *RUNX3* to influence EOC cell behavior. Importantly, we used both transcript variants namely TV1 expressed from promoter-1 and not affected by methylation and TV2 regulated by CpG island associated promoter-2.

### 2.1. Ectopic Expression of CaMKIINα and RUNX3 in EOC Cells

**Figure 1:**
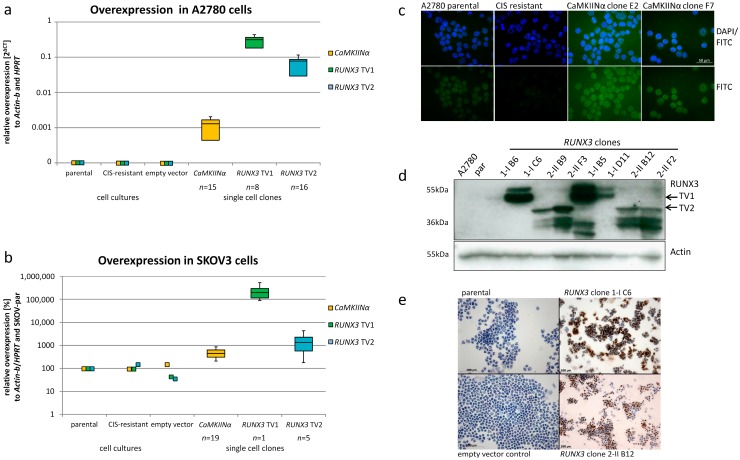
Overexpression of *CaMKIINα* and *RUNX3* TV1 and TV2 in A2780 and SKOV3 cells. (**a**) Boxplot showing the overexpression of the transgenes in A2780 cells compared to untransfected parental, cisplatin (CIS)-resistant cells and the empty vector controls expressing neither *CaMKIINα* nor *RUNX3*; (**b**) Boxplot showing the relative expression of the candidate genes in SKOV3 cells compared to the untransfected parental cells. Whereas CIS-resistant cells and the empty vector controls did not show an increased expression the transfected cells exhibited high levels of the transgenes. All data were normalized against the housekeeping genes *Actin-b* and *HPRT*; (**c**) Immunofluorescence staining for CaMKIINα exemplary for A2780 validating protein re-expression in transfected resistant A2780 clones (scale bar 50 µm); (**d**,**e**) Western blot and immunocytochemistry (scale bar 100 µm) confirming expression of differently sized RUNX3 isoforms in exemplary single cell clones. (Boxplots depict minimum and maximum values (whiskers); the inter quartile range (box) and the median (line)).

Single cell clones transfected with the pBK-*CaMKIINα* vector were screened for overexpression via quantitative RT-PCR with gene specific primers. A total of 19 SKOV3 and 15 A2780 single cell clones significantly overexpressing *CaMKIINα* were established (Figure 1a,b). Protein expression was analyzed by immunofluorescence staining showing reduced CaMKIINα levels in resistant cells and the re-expression in transfected clones (Figure 1c).

For *RUNX3*, altogether 6 SKOV3 and 24 A2780 single cell clones were distinguished. Variations in overexpression and cell heterogeneity between the different clones were already visible by immunocytochemical staining. Of note is that the αRUNX3 antibody recognizes an epitope located in the C-terminus and cannot differentiate between the isoforms. A distinction between transcript variants 1 and 2 was possible by Western Blotting and by quantitative reverse transcriptase-polymerase chain reaction (qRT-PCR) (Figure 1a,b,d). In contrast, TV1 overexpression resulted in the specific double band (RUNX3 isoform p46 and p46 with potential posttranslational modification [23]), TV2 translates into the shorter isoform p44 (upper band) and several smaller proteins (Figure 1d). Since the full-length transcript could be detected by RT-PCR, these may represent RUNX3 degradation products, or may arise from internal translation initiation processes. In SKOV3 cells, pBK-*RUNX3* transfection led to a 2- to 5000-fold overexpression compared to the untransfected parental SKOV3 cells. A pronounced overexpression was also accomplished in A2780 cells. The calculated fold changes of *RUNX3* overexpression in the two cells lines also correlated with the amount of positively stained cells using immunocytochemical scoring (Figure 1e). For functional assays we used single cell clones with different expression level thereby ensuring unbiased results (Supplementary Figure S1).

### 2.2. Cisplatin Sensitivity in CaMKIINα Overexpressing Resistant Cells

**Figure 2:**
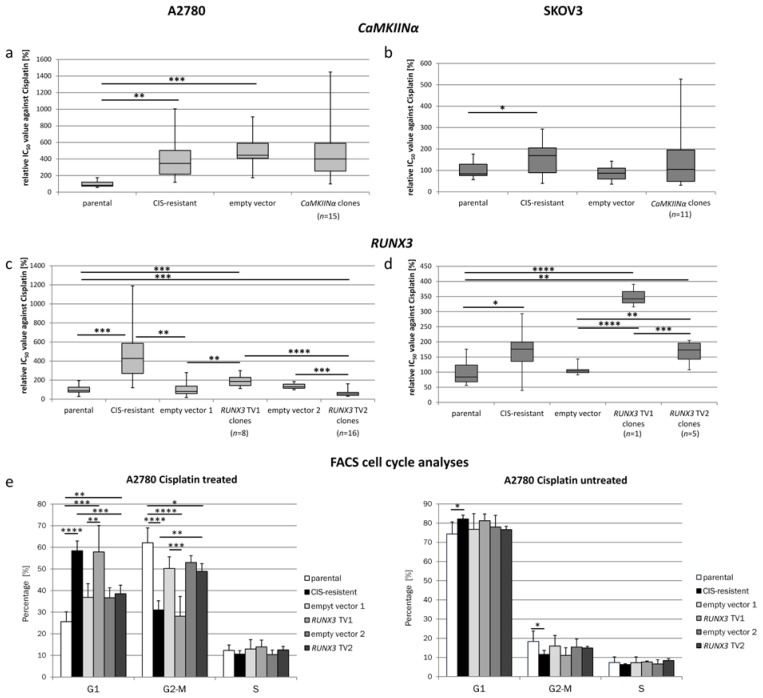
Sensitivity of the transgene expressing cells towards cisplatin. (**a**) IC_50_ measurements of A2780-CIS cells overexpressing *CaMKIINα*. The resistant A2780-CIS cells and the empty vector control presented the expected increased resistance towards cisplatin compared to parental A2780 cells. No significant change of IC_50_ values was observed when *CaMKIINα* was overexpressed in A2780-CIS; (**b**) In SKOV3-CIS cells the overexpression of *CaMKIINα* as well resulted in unchanged cisplatin sensitivity; (**c**) IC_50_ measurements of parental A2780 cells overexpressing *RUNX3* in its two transcript variants. A2780 single cell clones expressing *RUNX3* TV1 showed a significantly increased cisplatin resistance compared to the empty vector control and the parental cells but did not reach the resistance level of A2780-CIS cells. In contrast the A2780 *RUNX3* TV2 single cell clones presented a significantly increased sensitivity. When comparing the A2780 clones expressing *RUNX3* TV1 and clones expressing *RUNX3* TV2, the difference in sensitivity was highly significant; (**d**) SKOV3 cells expressing *RUNX3* TV1 exhibited a strong increase in cisplatin resistance while TV2 led to a slight increase, only. The difference between the cells expressing the two variants was significant; (**e**) Cell cycle analysis of A2780 cells revealed a significantly reduced G2/M arrest in cisplatin treated cells overexpressing *RUNX3* TV1, resembling resistant A2780 subcultures. Mean values are shown for different *RUNX3* overexpressing clones (*n* = 4). For all subfigures: * *p* < 0.05, ** *p* < 0.01, *** *p* < 0.001, **** *p* < 0.0001. (Boxplots depict minimum and maximum values (whiskers); the inter quartile range (box) and the median (line). Bar charts depict the mean ± SD).

When testing *CaMKIINα*-expressing resistant cells the mean IC_50_ value of single cell clones was not different from resistant cells or empty vector control clones (Figure 2a,b). Only a few individual single cell clones showed a significant difference to the control cells. The high variability of IC_50_ values for the single cell clones may point to a variable cisplatin sensitivity of single cells from resistant A2780 and SKOV3 cultures. This assumption is supported by the large variation of IC_50_ values for the resistant cultures itself. Furthermore, no difference in the *CaMKIINα* overexpression level was identified in groups of clones with IC_50_ values lower or higher than the IC_50_ of resistant cells excluding an effect of *CaMKIINα* (Supplementary Figure S2). All in all, the tested A2780 *CaMKIINα* expressing single cell clones exhibited an average IC_50_ value of 21 µM, similar to the one of the initially transfected resistant A2780 cells. Comparably SKOV3 single cell clones expressing *CaMKIINα* showed an average IC_50_ equal to the one of SKOV3-CIS cells—45 µM.

### 2.3. Cisplatin Sensitivity under the Influence of RUNX3

Transfecting *RUNX3* transcript variant 1 and 2 into the two respective ovarian cancer cells lines resulted in variable changes of cisplatin sensitivity. The strongest difference in the response towards cisplatin was measured in *RUNX3* transfected A2780 cells (Figure 2c). While *RUNX3* TV1 expressing A2780 clones had significantly higher cisplatin resistance levels, the expression of TV2 led to a significant reduction of the resistance compared to the empty vector control cells and parental A2780 cells. Comparing the IC_50_ value of the reference cells with the mean of the relative IC_50_ values of all tested clones expressing *RUNX3* TV1, a significant increase of the resistance was detected (187.1%, SD ± 52.8%, *p* < 0.01). Contrary, A2780 single cell clones expressing transcript variant 2 exhibited a significant decreased mean relative IC_50_ value of 61.9% (SD ± 31%; *p* < 0.001) compared to the reference cells (Figure 2c). To obtain first insights into underlying mechanisms, the cell cycle distribution of A2780 cells was analyzed by FACS experiments (Figure 2e). *RUNX3* TV1 expressing cells (clones #1-IC7, #1-ID11) did show a significantly reduced G2/M arrest under cisplatin treatment (28.1%) in comparison to parental (62.08%, *p* < 0.0001) or *RUNX3* TV2 expressing cells (48.89%, clones #2-IC11, #2-IIB9, #2-IIF10, #2-IIF12; *p* < 0.0001), but similar to resistant A2780 cells (31.02%; *p* > 0.05). In SKOV3 cells we did not detect contrary effects of the transcript variants and both increased the resistance compared to parental and empty vector controls (Figure 2d). Albeit *RUNX3* TV2 could not increase the sensitivity of SKOV3 cells, a different behavior of both transcript variants was identified. The cisplatin sensitivity of *RUNX3* TV2 expressing clones was significantly higher than that of the *RUNX3* TV1 clone (*p* < 0.001).

### 2.4. Reduction of Cellular Functions through CaMKIINα Overexpression

**Figure 3:**
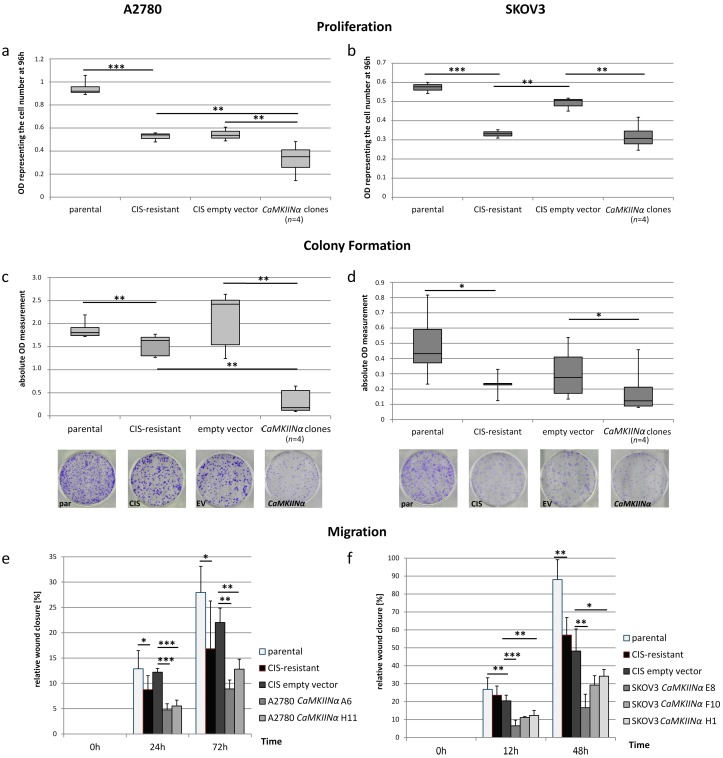
Effects of *CaMKIINα* overexpression on cellular functions. (**a**,**b**) The *CaMKIINα* expressing cells showed a significant reduction in proliferation compared to the empty vector. This was observed in A2780 as well as SKOV3 cells; (**c**,**d**) The ability to establish colonies independent from other cells was also evaluated in *CaMKIINα* expressing cells. The *CaMKIINα* clones established fewer colonies as the control cells. A significant reduction in this cellular property was seen in both cell lines. Pictures show exemplary staining of 6 cm dishes; (**e**) The overexpression of *CaMKIINα* resulted in a significant reduction of chemoattractant-independent migration of A2780. An overall reduction of migration by 41% was seen compared to the control cells; (**f**) In SKOV3 cells all tested *CaMKIINα* single cell clones presented a similar reduction of the migratory behavior as A2780. Two of three clones had a significant reduction in the migratory behavior. An overall reduction of 21% was observed. For all subfigures: * *p* < 0.05, ** *p* < 0.01, *** *p* < 0.001. (Boxplots depict minimum and maximum values (whiskers); the inter quartile range (box) and the median (line). Bar charts depict the mean ± SD).

In both cell lines the overexpression of *CaMKIINα* resulted in a decrease of the cellular functions measured by proliferation, migration, and colony-formation ability assays (Figure 3 and Supplementary Figures S3–S5). A strong and significant decline in proliferation was seen in the four tested *CaMKIINα* expressing A2780-CIS single cell clones compared to both A2780-CIS and empty vector clones. SKOV3-CIS clones with *CaMKIINα* showed a significant lower proliferation only if compared to the empty vector. Thus, *CaMKIINα* may affect SKOV3 proliferation, though not to the same extent as A2780 cells. However, SKOV3-CIS cells transfected with the empty vector exhibited an increased proliferation capacity compared to the resistant cells likely caused by the enrichment of proliferating cells during single cell cloning. In all other functional tests resistant cells and empty vector controls did not show significantly different results. The tested A2780 *CaMKIINα* single cell clones presented a very strong decline of the colony formation ability both compared to empty vector cells and A2780-CIS (Figure 3c, *p* < 0.01). In SKOV3 cells the overexpression of *CaMKIINα* also led to a minimized ability to form colonies (Figure 3d). Analyzing migration in a wound healing assay, the *CaMKIINα* expressing cells of both cell lines had a significant diminishment of the migratory ability after 24 h compared to single cell clones expressing the empty vector or the resistant cultures (*p* < 0.05). This difference further enlarged over time (Figure 3e,f).

### 2.5. Dualistic Effect of the RUNX3 Transcript Variants on Cellular Functions

**Figure 4:**
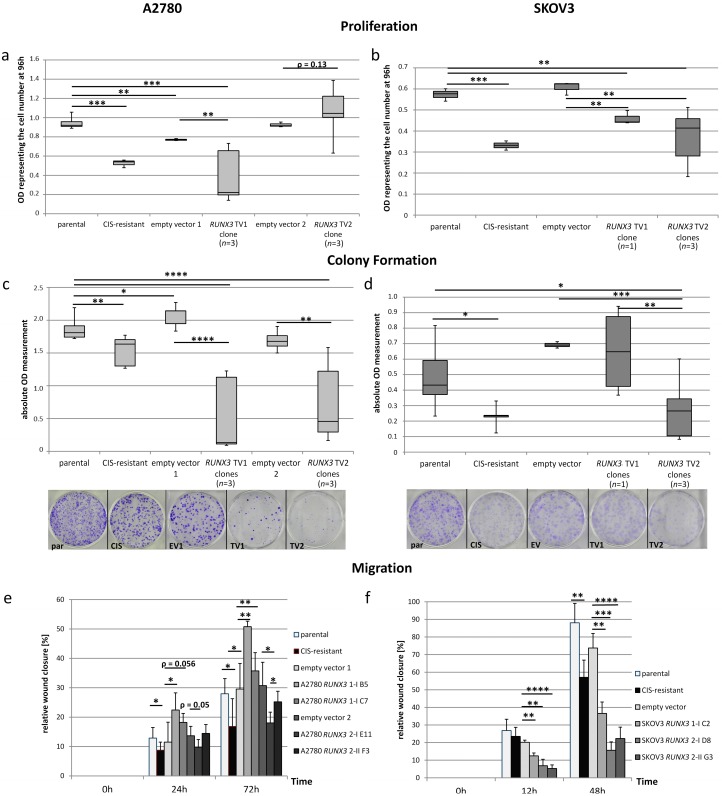
Effects of *RUNX3* overexpression on cellular functions. (**a**,**b**) In A2780 cells, the expression of the two transcript variants resulted in different proliferation rates. While variant 1 led to a high decline, variant 2 caused an increase in the proliferation. In SKOV3 cells the two *RUNX3* transcript variants led both to a reduction of proliferation; (**c**,**d**) The overexpression of *RUNX3* transcript variant 1 in A2780 cells led to a reduction while in SKOV3 cells the tested clone showed no change in its colony formation ability. All single cell clones of both cell lines expressing *RUNX3* TV2 showed significantly reduced colony formation compared to the parental or control cells. Because of space limitations the exemplary colony formation picture from 6 cm dishes for A2780 empty vector 2 was excluded; (**e**) The overexpression of *RUNX3* TV1 in A2780 resulted in a significant increase of migration while a reduction was seen in clones expressing *RUNX3* TV2. An overall reduction of migration by 14.8% was seen compared to the control cells; (**f**) In SKOV3 cells all tested *RUNX3* clones of both variants presented a decreased migratory behavior while the effect of transcript variant 1 was less pronounced. For all subfigures: * *p* < 0.05, ** *p* < 0.01, *** *p* < 0.001, **** *p* < 0.0001. (Boxplots depict minimum and maximum values (whiskers); the inter quartile range (box) and the median (line). Bar charts depict the mean ± SD).

*RUNX3* variants exhibited different properties depending on the cell line and functional read out. Whereas TV1 inhibited proliferation in both cell lines, SKOV3 cell proliferation was additionally inhibited by TV2 (Figure 4a,b). Contrary, A2780 cell proliferation was slightly but not significantly increased by transcript variant 2 proving potential differences in RUNX3 isoform functions. The colony forming capacity was affected differently by the transcript variants in SKOV3 but not A2780 cells (Figure 4c,d). Both variants inhibited A2780 colony formation significantly whereas only TV2 reduced SKOV3 colony number. The migratory movement differed between the variants (Figure 4e,f and Supplementary Figure S3). In A2780 cells as well as SKOV3 cells overexpression of *RUNX3* TV2 resulted in a significantly reduced migration compared to the empty vector control cells (*p* < 0.05). *RUNX3* TV1 on the other hand, led to a significant increase of the migratory behavior of A2780 compared to the reference indicating a promoting effect of transcript variant 1. In the case of SKOV3 cells, TV1 inhibited migration but to a smaller extent than TV2.

### 2.6. In-Silico Analysis of Published Datasets for RUNX3

**Figure 5:**
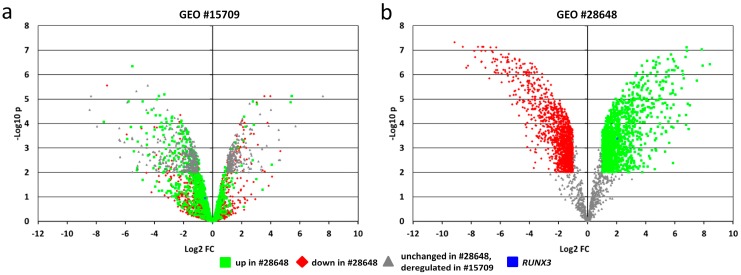

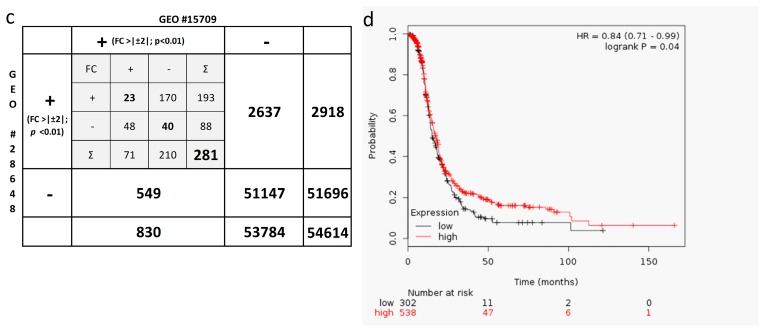
Cisplatin resistance in A2780 can be associated with different genome wide gene expression profiles. (**a**,**b**) Volcano plots for transcripts differentially expressed between parental A2780 and cisplatin-resistant A2780 (*p* < 0.01 and fold change (FC) >2 or <−2) from either GEO#15709 (**a**) or GEO#28648 (**b**). Color codes relate to data from GEO#28648 depicting upregulated genes (green), down regulated genes (red) or genes not differentially expressed in this dataset, but in GEO#15709 (grey). The comparison of both plots indicates largely different genome wide expression changes during resistance development in the two independent resistant A2780 cell cultures (GEO#15709; GEO#28648); (**c**) Cross table comparing the number of targets which are differentially expressed or unchanged in both data sets (outer table). Whereas most genes were not affected in both sets (93.7%) only 281 targets from 2918 (GEO#28648) or from 830 (GEO#15709) are concordantly deregulated. Moreover, only 63 from these 281 transcripts are deregulated in the same direction (23 upregulated, 40 down regulated; inner table, grey). The expression of the majority of 218/281 transcripts is changed in opposite directions; (**d**) Prognostic value of *RUNX3* gene expression analyzed by the Kaplan–Meier-Plotter for public available datasets from EOC patients (serous, FIGO III/IV, treated with platinum; *n* = 840). Patients with high *RUNX3* gene expression show slightly increased progression free survival (PFS) compared to the *RUNX3*-low group.

RUNX3 is described as mediating platinum resistance in A2780 cells [24]. Our data point to transcript variant 1 in mediating this effect (Figure 2c) but we did not observe an aberrant *RUNX3* expression in resistant A2780 (Figure 1). Therefore, we have interrogated public data (NCBI Geo database, TCGA) partly using the Kaplan–Meier Plotter tool [25]. Two genome-wide gene expression datasets (U133+2.0 Affymetrix platform) of independent isogenic pairs of A2780 platinum-sensitive and -resistant subcultures (GSE#15709 [26], GSE#28648 [27]) are available. We are not aware of other ovarian cancer cell lines with independent isogenic platinum-resistant subcultures enabling an analysis of the heterogeneity of resistance states. The comparison of both A2780 data sets clearly proves the presence of at least two largely different genome expression states associated with resistance (Figure 5a–c). The overall number of deregulated transcripts (fold change >|±2|; *p* < 0.01 BH-corrected) differed 3.5-fold (2918 vs. 830 transcripts) and only 281 transcripts were deregulated in both resistant A2780 cultures. Only the minority of these 281 transcripts were deregulated in identical direction in both isogenic pairs (63/281). *RUNX3* was upregulated in GSE#28648 contributing to platinum resistance in these cells [24]. Thus, the existence of two platinum resistance states for A2780 of which only one depends on *RUNX3* (potentially TV1) can be supposed. Unfortunately, array-based gene expression analyses cannot differentiate between *RUNX3* transcript variants. Variable effects of *RUNX3* in independent cultures of A2780 may also translate into variable associations between *RUNX3* expression and clinical outcome in different patient cohorts. Barghout et al. analyzed GSE#28739 [28] and detected an up-regulation of *RUNX3* in platinum-resistant EOC consistent with their A2780 data [24]. We cannot confirm these data by in-silico analyses (Kaplan−Meier Plotter, Figure 5d). Stratification of EOC patients (serous, FIGO III/IV, treated with platinum; *n* = 840) by *RUNX3* expression revealed that patients with high *RUNX3* expression have slightly improved progression-free survival.

## 3. Discussion

Since the two analyzed genes play a crucial role in cellular functions, *CaMKIINα* and *RUNX3* are highly regulated in normal healthy cells. The hypermethylation of at least one of both genes is associated with reduced progression-free survival, implicating a functional contribution to tumor properties [13]. Thus, we aimed to analyze both genes together, albeit we had no data pointing to a functional relationship between the two proteins. Whether these two genes have a functional relevance and influence ovarian carcinogenesis was not fully elucidated so far. Only the function of *CaMKIINβ* was evaluated in ovarian cancer [17], but there is evidence that both endogenous CaMKII inhibitors function in a comparable way. The definitive proof for this hypothesis was not yet given. There is extensive literature on *RUNX3* for various tumor entities and it appears to be causally involved in tumor development [29]. Interestingly, no one so far has rigorously evaluated the functional difference between the two transcript variants in carcinogenesis; even though it is known that the variants are differentially regulated, and resulting proteins differ in their N-terminus.

After initial surgery the platinum based chemotherapeutics carbo- or cisplatin are used as standard treatment [8,9,10]. Consequently, an important aim of the present study was to evaluate the influence of *CaMKIINα* or *RUNX3* overexpression on the cisplatin sensitivity of EOC cells. Overexpressing *CaMKIINα* did not result in an effect on cisplatin sensitivity or in a correlation between *CaMKIINα* expression level and IC_50_ values. This might have been expected, at least for SKOV3 cells, because of the resistance-associated hypermethylation of *CaMKIINα* in this cell line [13]. From this result, we conclude that the resistance acquisition in SKOV3 is not a direct consequence of the *CaMKIINα* promoter methylation that can be reverted solely by overexpression of *CaMKIINα*. Nevertheless, we cannot exclude a contribution of *CaMKIINα* at early stages of resistance development or a function in regulatory mechanisms, incompatible with a dominant resistance phenotype. The reduced proliferation and colony-forming ability after *CaMKIINα* overexpression in resistant cells may point to such an involvement. Moreover, treatment of ovarian cancer cells with calmodulin inhibitors increases the sensitivity to cisplatin [30,31]. Thus, the contribution of Ca^2+^-calmodulin pathways to cisplatin sensitivity should still be investigated further. In the functional assays we demonstrated that *CaMKIINα* drives the cells towards a loss of oncogenic behavior. A reduction in proliferation (A2780 only) and reduced colony formation were seen, indicating a tumor suppressive function of *CaMKIINα*. Comparable effects were detected in prostate cancer cells by Wang and colleagues [16]. Furthermore, the influence of CaMKII and its endogenous inhibitors on proliferation and cellular growth was also investigated in various other tumor entities pointing in the same direction [17,32,33,34,35,36]. The endogenous CaMKII inhibitors possibly lead to a cell cycle arrest in S phase [36]. The reduction of migratory behavior induced via *CaMKIINα* overexpression was not observed so far, but indicates in line with the other results that *CaMKIINα* can act tumor suppressive in ovarian cancer cell lines. This matches studies showing a protective role of this gene from developing a more aggressive and extensive thyroid tumor [18].

In contrast to *CaMKIINα*, we see direct effects on the cisplatin sensitivity when overexpressing *RUNX3* transcript variants in the analyzed ovarian cancer cell lines. Both *RUNX3* variants led to a gain of resistance but with a largely different effect size in SKOV3 cells. Whereas TV 1 increased the resistance of SKOV3 3.5-fold, TV 2 induced a 1.16-fold resistance only. In A2780 the resistance-effect was only seen when transfected with transcript variant 1 (1.9-fold increase of IC_50_). Moreover, the cells became sensitive against cisplatin when overexpressing *RUNX3* TV2 (2.3-fold decreased resistance). The contrary effects of both transcript variants are associated with different cell cycle distributions under cisplatin treatment i.e., *RUNX3* TV1 reduced the G2/M arrest to a level comparable with resistant A2780 cells (Figure 2e). The 1.9-fold increased resistance of A2780 after transfection of *RUNX3* TV1 resembles data from Barghout et al. showing a 1.8-fold increased resistance for carboplatin treatment of *RUNX3* overexpressing A2780 [24]. The different behavior of A2780 and SKOV3 after *RUNX3* TV2 overexpression is maybe related to the *p53* status (A2780 *p53*wt; SKOV3 *p53*-null). RUNX3 acts as co-activator of p53 in DNA-damage response and apoptosis induction [37]. Our data point towards *RUNX3* TV2 in mediating this effect. Combining the herein presented cell line data with the clinical data from our study [13], we hypothesize that the cisplatin sensitivity-mediating transcript variant 2 is inactivated via P2 promoter methylation—enabling a shift to transcript variant 1 and causing a platinum-resistant phenotype. Thus, the resistance may lead to a worsened outcome for the patient. It was already described that the response to platinum-based chemotherapy is influenced by the deregulation of RUNX proteins or Abl-YAP1-p73 signaling [23], suggesting the importance of the transcription factors for the therapy’s success. Beside platinum resistance, we measured *RUNX3* transcript-specific effects on cell proliferation and migration. While growth of the analyzed cell lines was reduced by overexpression of *RUNX3* TV1 the same anti-proliferative effect with overexpression of *RUNX3* TV2 was just seen in SKOV3 cells. The slight induction of proliferation in A2780 cells expressing variant 2 is also a plausible explanation for the fact that those cells experience higher cisplatin sensitivity. Already Nevandunsky and colleagues reported the effect that *RUNX3* overexpression leads to an increased cellular proliferation of A2780 [38]. Since no differentiation between the two transcript variants was done they assumed a general oncogenic function for *RUNX3*. But it appears that a faster proliferation in A2780 is just seen in *RUNX3* TV2 expressing cells with concomitantly increased platinum sensitivity. The net effect may not provide the cancer cells with a survival benefit after chemotherapy in vivo. The cisplatin-resistant *RUNX3* TV1-expressing A2780 cells presented also an increased migratory movement further underscoring the hypothesis of *RUNX3* TV1 as mediating an aggressive phenotype. Additionally, murine leukemia virus integration in the *RUNX3* loci, resulting in overexpression of P1 derived *RUNX3* TV1, caused lymphomas, and revealed a potential oncogenic function [39]. The difference in the observed aggressiveness between cells overexpressing *RUNX3* variants is possibly caused by the different ways the two transcript variants regulate the NF-κB pathway. The oncogenic activity of NF-κB is enhanced by the action of *RUNX3* TV1 encoded isoforms while TV2 impacts its cancer inhibitory function [20]. Combining this with the fact that NF-κB induces the migration of immune cells [40], one may have a potential explanation why *RUNX3* TV1 and TV2 transfected cells differ in their migratory properties. Altogether, the crucial factor in patients’ tumors may not be the proliferation rate (TV2-mediated), but the ability to persist unaffected by the chemotherapeutic drug and the ability to form metastasis (TV1-mediated). Thus *RUNX3* TV1 may favor tumor progression and relapse.

Our data provide novel insight on the functional roles for RUNX3. RUNX3 is described to exert tissue-specific functions leading to both oncogenic and tumor-suppressive roles depending on analyzed tumor entities [23]. Moreover, RUNX3 can act in both ways within pancreatic cancer cells depending on cellular background and functional read out [41]. Unfortunately, most papers analyzing RUNX3 function or aberrations neither differentiate between isoforms, nor specify which transcript variant is analyzed. Such differentiation is hampered by the absence of specific antibodies against the protein isoforms, and a small difference in the 5′ nucleotide sequence only. Thus, we cannot estimate the contribution of the isoforms to the reported variable roles of RUNX3 in general. Even for the platinum resistance phenotype, both transcript variants and cellular background differences may contribute to contrary RUNX3 effects. We have shown dichotomous roles for *RUNX3* transcript variants in A2780 and our results for *RUNX3* TV1 correspond to results from Barghout and colleagues [24]. They describe the contribution of RUNX3 to platinum resistance in A2780 but do not report about the transcript specificity of their results. Overexpression of *RUNX3* in A2780 resulted in increased resistance, whereas knockdown in A2780CP lead to higher sensitivity of this resistant subculture [24]. Nevertheless, we did not detect an overexpression of *RUNX3* in resistant cells but our in-silico analysis proves the presence of at least two different genome-wide expression states, associated with platinum resistance in A2780, of which only one is related to *RUNX3* overexpression (Figure 5). Thus, the herein analyzed A2780-CIS cells may represent the second resistance state not depending on *RUNX3*, explaining the absent up-regulation of *RUNX3* (Figure 1). Analyses of RUNX3 expression as biomarker in clinical samples is hampered by missing isoform-specific antibodies and a frequent transcript quantification not differentiating between variants. Additionally, the number of tumor-infiltrating immune cells influences the measured tissue-based *RUNX3* gene expression because of an inherent *RUNX3* expression in immune cells. Thus, potential bias can be induced because the number of immune cells itself has also prognostic effects for ovarian cancer patients [42]. These reasons may contribute to observed discrepancies between publicly available datasets (Figure 5d and Barghout et al. [24]).

Limitations of the present study are the analysis of single cell clones and the choice of used cell lines. The necessity for using cell clones arose because of a high frequency of transgene negative cells after selection precluding the analysis of batch cultures. Nevertheless, we reduced sources of bias by analyzing clones with different transgene expression level (clonal effects) and empty vector controls for each transfection experiment (batch effects, A2780 *RUNX3* transfection). The functional implications of *CaMKIINα* and *RUNX3* expression in ovarian carcinogenesis were analyzed using A2780 and SKOV3 cells. It was shown that both cell lines do not represent high-grade serous EOC well [43]. Thus, data from this study may not be completely transferred to all high-grade serous EOC, but main conclusions fit to previously published methylation data, and may partially explain the worse clinical outcome for ca. 40% of type II EOC patients with PFS <3 years [13]. We had chosen these lines for functional analyses because of the availability of isogenic pairs of sensitive and resistant cultures in our lab and the fact that both cell lines were the ones most often used in published studies [43]. Specifically most data about RUNX3 and CaMKIINβ functions in EOC were derived from A2780 or SKOV3 [17,22,24,38].

Applying various functional assays, we prove that *CaMKIINα* acts as a tumor suppressor, as already seen in other cancer types [16,36]. Both endogenous CaMKII inhibitors operate in ovarian cancer in the equal manner [17]. Likewise, this evidence is supported by the fact that *CaMKIINα* promoter hypermethylation is associated with poor patient’s prognosis [13]. Evaluating the data of the second marker gene, we demonstrated that *RUNX3* TV1 has a more aggressive potential than the shorter variant 2. In accordance with clinical data showing promoter P2 hypermethylation in patients with poor prognosis [13], *RUNX3* TV2 can be labelled as the more tumor suppressive version. Therefore, we encourage the idea that RUNX3 has a dualistic effect on EOC cells as already postulated by Chuang and Ito [19]. Because the two RUNX3 protein isoforms function in a different manner the necessity to discriminate between the two transcript variants can be deduced from this study. So far such a distinction was not regularly considered when evaluating its role in carcinogenesis. In summary, our data showed that *CaMKIINα* and *RUNX3* are functionally involved in ovarian carcinogenesis and support that the methylation of both genes should be further evaluated as biomarker.

## 4. Materials and Methods

### 4.1. Cell Culture

SKOV3 and A2780 ovarian cancer cells were maintained in RPMI (Roswell Park Memorial Institute) Medium 1640—GlutaMAX™-I (Life Technologies, Darmstadt, Germany) containing 10% fetal calf serum and 1% Pen Strep (Life Technologies) in a 37 °C incubator under 5% CO_2_ and 95% humidity. Respective cisplatin-resistant cells of each cell line were generated as published for SKOV3 [13]. Transfected cells were cultured in cell line specific selection RPMI media comprising 0.3 or 0.4 mg/mL of the selection antibiotic Geneticin—G418 for A2780 and SKOV3 respectively. Cell lines were obtained from A. Brüning (Munich, Germany, A2780) and P. Altevogt (Heidelberg, Germany, SKOV3). Cell line authentication was done by STR profiling in September 2014 and February 2016 and revealed the correct identity depicted by a 97 % and 100 % match for A2780 and SKOV3 (ICLAC Match Criteria Worksheet [44]) compared to STR reference profiles (ATCC and [45]).

### 4.2. Transfection and Single Cell Clone Generation

The copy DNA (cDNA) of *CaMKIINα* (NM_018584) and *RUNX3* in its transcript variants 1 (NM_001031680) and 2 (NM_004350) were cloned into a pBK-CMV expression vector flanking *Bam*HI and *Xba*I sites by PCR-amplification of the open reading frames from cDNA of the ovarian cancer cell line HOC-7 (*CaMKIINα*) or a biopsy from an ovarian cancer patient (#2340; *RUNX3*). Correct, error-free sequences were confirmed by Sanger-sequencing of plasmid DNA. A2780 cells were transfected using the IsiFECT™ transfection reagent (BianoScience, Zwickau, Germany) while the Lipofectamine transfection reaction (Invitrogen, Darmstadt, Germany) was used for SKOV3 cells. Empty vector pBK-CMV transfected cells were used as controls. The *RUNX3* transcript variants were transfected into A2780 in different experiments resulting in two different control cell cultures (empty vector 1 and 2). The pBK-*CaMKIINα* plasmids were transfected into cisplatin-resistant cells while the pBK-*RUNX3* plasmids were transfected into parental cells. To generate single cell clones the cells were either seeded in a 96-well plate with statistically less than one cell per well or seeded with 500 cells in a 10 cm Petri dish and directly picked after colony formation. The cells were expanded in different dishes till they were able to cover a 6 cm Petri dish. From the 6 cm Petri dish, cells were cryogenic stored or treated with lysis buffer for RNA isolation (Macherey Nagel, Düren, Germany).

### 4.3. Immunocytochemical Staining Assays

Up to 30,000 cells of each single cell clone were grown for 24 h in one well of a 8-well chamber slide (Nalgene Nunc International, Penfield, NY, USA) and used for the immunocytochemical staining against *RUNX3* as previously described [13]. The overexpression of *RUNX3* was confirmed via qRT-PCR for each positively stained clone. CaMKIINα protein expression was analyzed by immunofluorescence analysis as published [13].

### 4.4. Gene expression Analysis

To analyze the overexpression of transfected genes expression analyses were conducted using established techniques and gene specific primers (Table 1) [13].

### 4.5. Western Blot Analysis

Total proteins were extracted using RIPA (Radioimmunoprecipitation assay) buffer supplemented with protease inhibitors (Complete mini, Roche, Mannheim, Germany). Equal amounts of proteins from each lysate were separated on a 10% sodium dodecyl sulfate-polyacrylamide gel electrophoresis (SDS-PAGE) and transferred onto a polyvinylidene fluoride (PVDF) membrane (Millipore, Burlington, MA, USA). The Tris-buffered saline with Tween-20 (TBST)-blocked membrane was incubated with the appropriate primary antibody overnight at 4 °C; RUNX3 (R3-5G4, Santa Cruz Biotechnology, Dallas, TX, USA), Actin (BD Biosciences, San Jose, CA, USA). After being washed three times in 1xTBST the membrane was exposed to the secondary antibody against mouse IgG (Jackson Laboratories, Bar Harbor, ME, USA) for 1 h at room temperature (RT). The protein expression was visualized by chemiluminescence using ECL reagents (Pierce, Thermo Fisher Scientific, Waltham, MA, USA). Actin was used as loading control.

### 4.6. IC_50_ Determination Against Cisplatin

A total of 1 × 10^4^ cells were seeded as technical triplicate in a 96-well plate in a total of 200 µL RPMI medium and cultivated for 24 h. Different cisplatin concentrations (between 100 and 0.1 µM) were generated through a serial dilution of a 1 mg/mL stock solution. The cells were incubated for 48 h under the substances influence. Afterwards the cells were incubated for 1 to 1.5 h with fresh RPMI medium before 15 µL MTT (tetrazolium dye 3-(4,5-dimethylthiazol-2-yl)-2,5-diphenyltetrazolium bromide) reagent (Promega, Mannheim, Germany) was added to each well and cells were lysed after incubation for 1 h. The emerging purple precipitate was measured at 570 nm using a microplate reader (SPECTROstar Omega, BMG Labtech, Freiburg, Germany). After background subtraction relative values were calculated and analyzed via GraphPad 5.0 software using non-linear regression analyses.

### 4.7. Cell Proliferation Assay

A total of 2500 cells were seeded as technical triplicates in a 96-well plate in a total of 100 µL RPMI medium. To assess the proliferation rate the CellTiter 96^®^ Non-Radioactive Cell Proliferation Assay (MTT) from Promega was used. Similar to the IC_50_ determination the cells were treated with 15 µL MTT reagent and incubated 1 h at 37 °C and lysed before spectrometric measurement at 570 nm. The amount of cells was analyzed after 24, 48, 72 and 96 h. To exclude variations in cell number an initial measurement was taken 2 h after seeding, and subtracted from all further measurement. To confirm the proliferation rate assessed with the MTT reagent and to exclude variations in the enzymatic activity between different cells one radioactivity based tritium (^3^H) assay was additionally performed.

### 4.8. Colony Formation Assay

Five hundred cells were seeded as a technical triplicate in 6-well plates and cultured in 2 mL RMPI medium for 10 days. The established colonies were washed with DPBS +/+ and fixed 20 min at 4 °C with ice-cold 70% EtOH. After washing with double-deionized water the fixed colonies were air-dried and stained with differently concentrated crystal violet solution for 5 min at RT. To stain A2780 cells a 0.1% crystal violet solution was used while for SKOV3 cells a 1% solution was needed to improve visible staining. Acetic acid (10% concentration) was used to extract the crystal violet pigments from the stained colonies and the amount of crystal violet was then measured at a wavelength of 630 nm. The measured optical density correlated with the amount of cells stained and therefore also with the numbers of colonies counted.

### 4.9. Migration-Scratch Assay

A total of 1 × 10^6^ (SKOV3) or 2.5 × 10^6^ (A2780) cells were seeded in a 6-well plate and grown in normal RPMI medium till 80–90% confluence was reached. Three scratches were introduced into the cell monolayer and washed with DPBS +/+ to remove cell debris. To measure just the migratory ability the cells were further cultivated in serum-free medium. Images were taken at three pre-defined areas of the scratches at the time points 0, 6, 12, 24 and 48 h. Since A2780 cells migrate slower, an additional image was taken 72 h after the scratch introduction. Analysis was then conducted using the open source software T-Scratch. The mean wound closure at the three pre-defined areas represented the result for each independent experiment.

### 4.10. Flow Cytometry

For cell cycle analysis cells were treated with 3 µM cisplatin for 48 h, washed twice with PBS, and fixed in 50% ice cold ethanol at −20 °C for at least 3 h. After washing out the ethanol with double-deionized water, cells were treated with RNase A and stained with propidium iodide (PI, 50 µg/mL). Cells were measured using a Canto II (BD Biosciences). Data were analyzed with FlowJo software (FlowJo, Ashland, OR, USA).

### 4.11. Statistical Analysis

Statistical comparisons of the data from the in vitro assays were conducted applying the Student *t*-test. *p*-values smaller than 0.05 were considered as statistical significant. All data are reported as mean ± standard deviation reflecting at least three independent experiments (*n* ≥ 3) for cell cultures or individual single cell clones. Averaged data for multiple clones represent the mean from individual clones gathered from at least three independent experiments. Boxplots depict minimum and maximum values (whiskers); the inter quartile range (box) and the median (line).

## 5. Conclusions

In vitro analyses of *CaMKIINα* and *RUNX3* identified an influence on proliferation, migration or platinum resistance consistent with a less aggressive phenotype in vivo for tumors without hypermethylation of these genes. Thus, the results support the association of hypermethylation and a worse prognosis for ovarian cancer patients. Importantly, *RUNX3* variants exhibited dichotomous roles, and studies analyzing *RUNX3* function should discriminate between them.

## Figures and Tables

**Table 1:** Primer data.

Primer	Sequence	Product Size & Annealing Temperature
*CaMKIINα*-F	5′-GACACCAACAACTTCTTCGG-3′	84 bp, 58 °C
*CaMKIINα*-R	5′-CAATAACAACCCGCTTGCT-3′	
*HPRT*-F	5′-ACGAAGTGTTGGATATAAGC-3′	214 bp, 56 °C
*HPRT*-R	5′-ATAATTTTACTGGCGATGTC-3′	
*ACTB*-F	5′-GGACTTCGAGCAAGAGATGG-3′	294 bp, 56 °C
*ACTB*-R	5′-GCAGTGATCTCCTTCTGCATC-3′	
*RUNX3*-TV1-F	5′-ATGGCATCGAACAGCATCTTC-3′	337 bp, 60 °C
*RUNX3*-TV1-R	5′-CCAATGCCACCACCTTGAA-3′	
*RUNX3*-TV2-F	5′-ATGCGTATTCCCGTAGACCC-3′	295 bp, 60 °C
*RUNX3*-TV2-R	5′-CCAATGCCACCACCTTGAA-3′	

## Supplementary Materials

The following are available online at www.mdpi.com/1422-0067/19/1/253/s1.

## Abbreviations

EOC	epithelial ovarian cancer
IC	inhibitory concentration
TV	transcript variant
5yr-SR	5-year-survival-rate
