# Supplementary material for: Functional Analyses of RUNX3 and CaMKIINα in Ovarian Cancer Cell Lines Reveal Tumor-Suppressive Functions for CaMKIINα and Dichotomous Roles for RUNX3 Transcript Variants
Source: Int J Mol Sci. 2018 Jan 15;19(1):253. doi: 10.3390/ijms19010253 (PMC5796200; doi:10.3390/ijms19010253)
Supplement: Supplementary file 1 [file ijms-19-00253-s001.pdf]

Functional analyses of *RUNX3* and *CaMKIINα* in ovarian cancer cell lines reveal tumor-suppressive functions for *CaMKIINα* and dichotomous roles for *RUNX3* transcript variants.

Karolin Heinze, Daniel Kritsch, Alexander Mosig, Matthias Dürst, Norman Häfner and Ingo B. Runnebaum

Department of Gynaecology, Jena University Hospital – Friedrich Schiller University, Jena, Germany

Supplementary Figures S1-S5

Figure S1

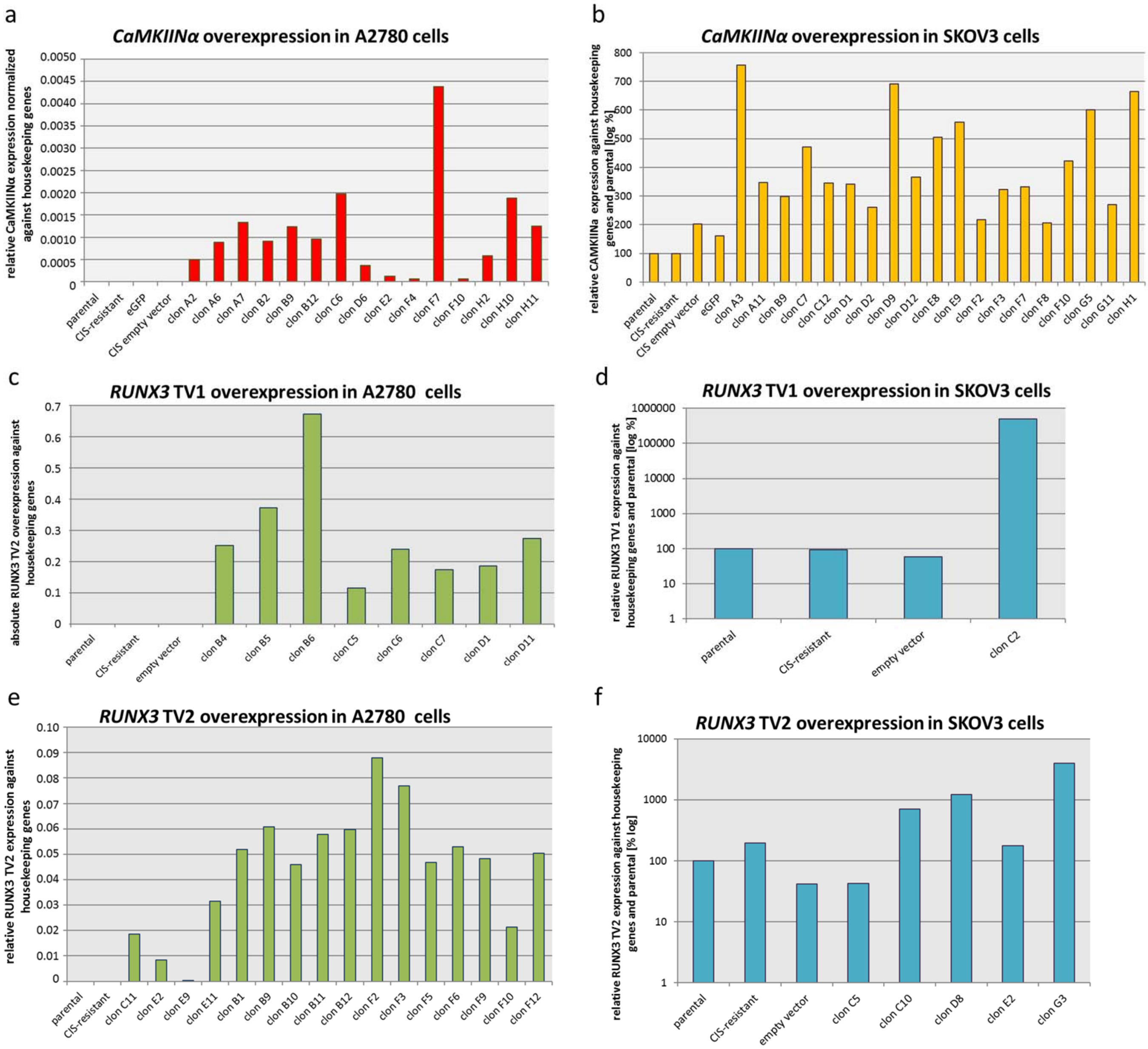

Supplementary Figure S1. Gene expression of the transgenes in A2780 and SKOV3 cells normalized against the housekeeping gene *Actin b* and *HPRT*. SKOV3 data are additionally normalized to the parental cells. (a) Relative expression of *CaMKIINα* in parental, CIS-resistant A2780 cells and *CaMKIINα* clones. (b) Relative expression of *CaMKIINα* in parental, CIS-resistant SKOV3 cells and the *CaMKIINα* clones showing an overexpression above the control cells. (c, e) Relative level of *RUNX3* TV1 (c) and TV2 (e) in A2780 cells confirmed an overexpression compared to parental and CIS-resistant cells. (d, f) Relative expression of *RUNX3* TV1 (d) and TV2 (f) in SKOV3 cells compared to parental, CIS-resistant and empty vector control cells.

Figure S2

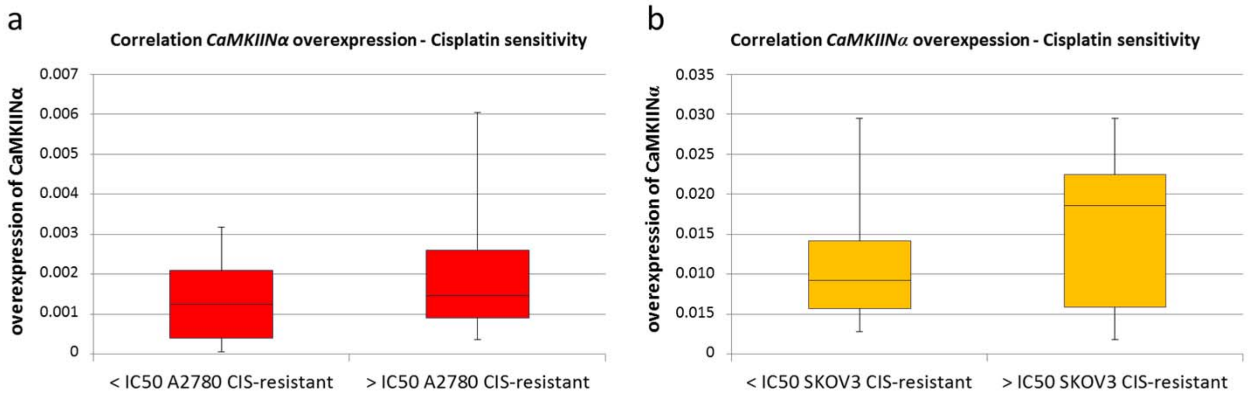

Supplementary Figure S2. Correlation of the *CaMKIINα* overexpression and the cisplatin sensitivity in A2780 (a) and in SKOV3 cells (b). No association between the responsiveness towards cisplatin and *CaMKIINα* level was seen.

Figure S3

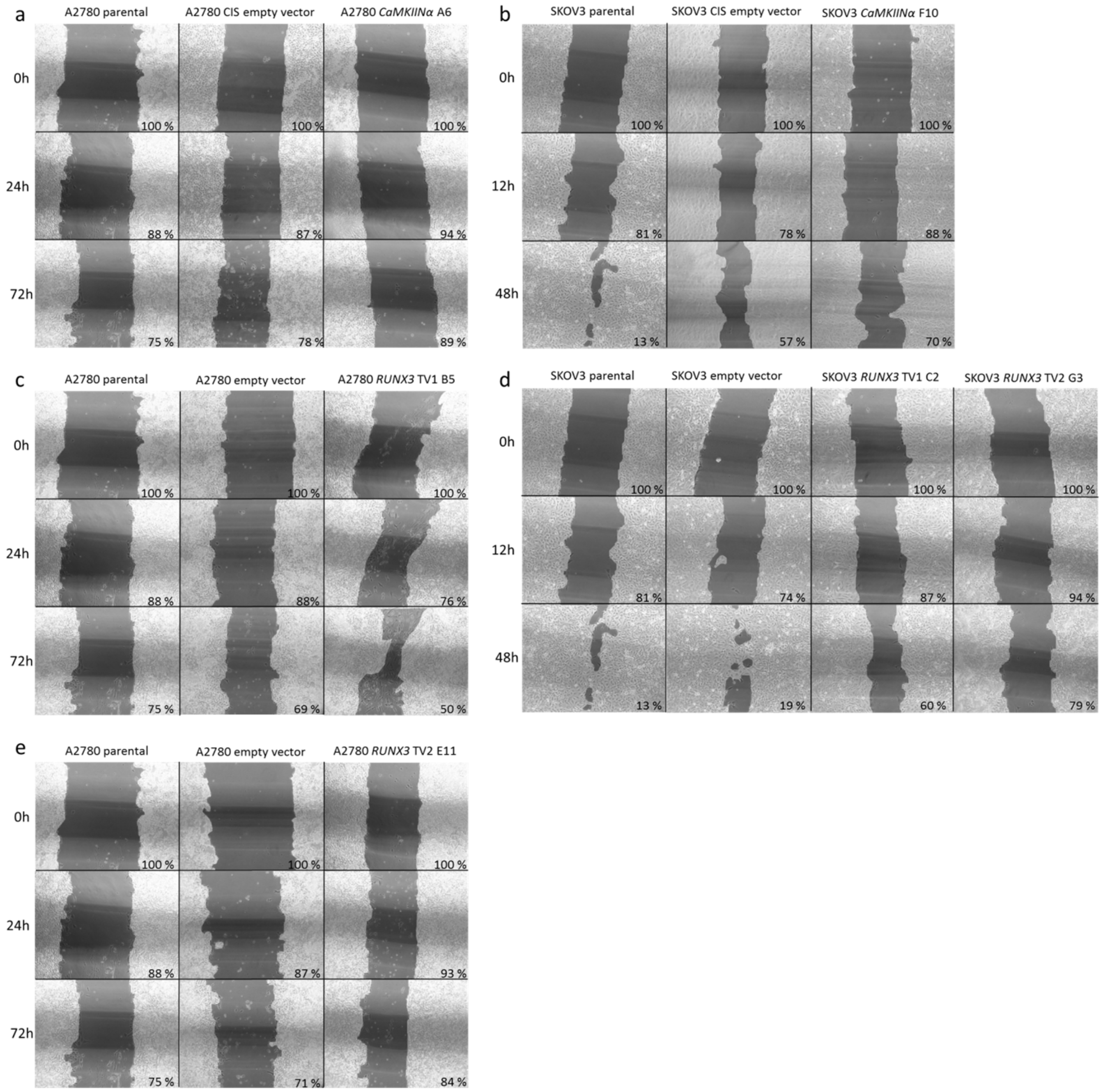

Supplementary Figure S3. Exemplary pictures of the migratory behavior of cells under *CaMKIINα* and *RUNX3* expression in the wound healing assay. Parental cell data for each cell line are identical for all 3 transcripts analyzed. Overview of observed migratory properties in A2780 cells (a) and SKOV3 cells (b) under *CaMKIINα* expression. A reduced wound closure was achieved in *CaMKIINα* single cell clones compared to control cells. The A2780 cells expressing *RUNX3* TV1 (c) showed an increase in the cellular migration while A2780 cells expressing *RUNX3* TV2 (e) experienced an inhibition of migration. In SKOV3 cells (d) both transcript variants led to a reduction of the wound closure but a distinct difference between the two variants was observed. In concordance to A2780 data *RUNX3* TV2 reduced the migratory ability stronger than TV1.

Figure S4

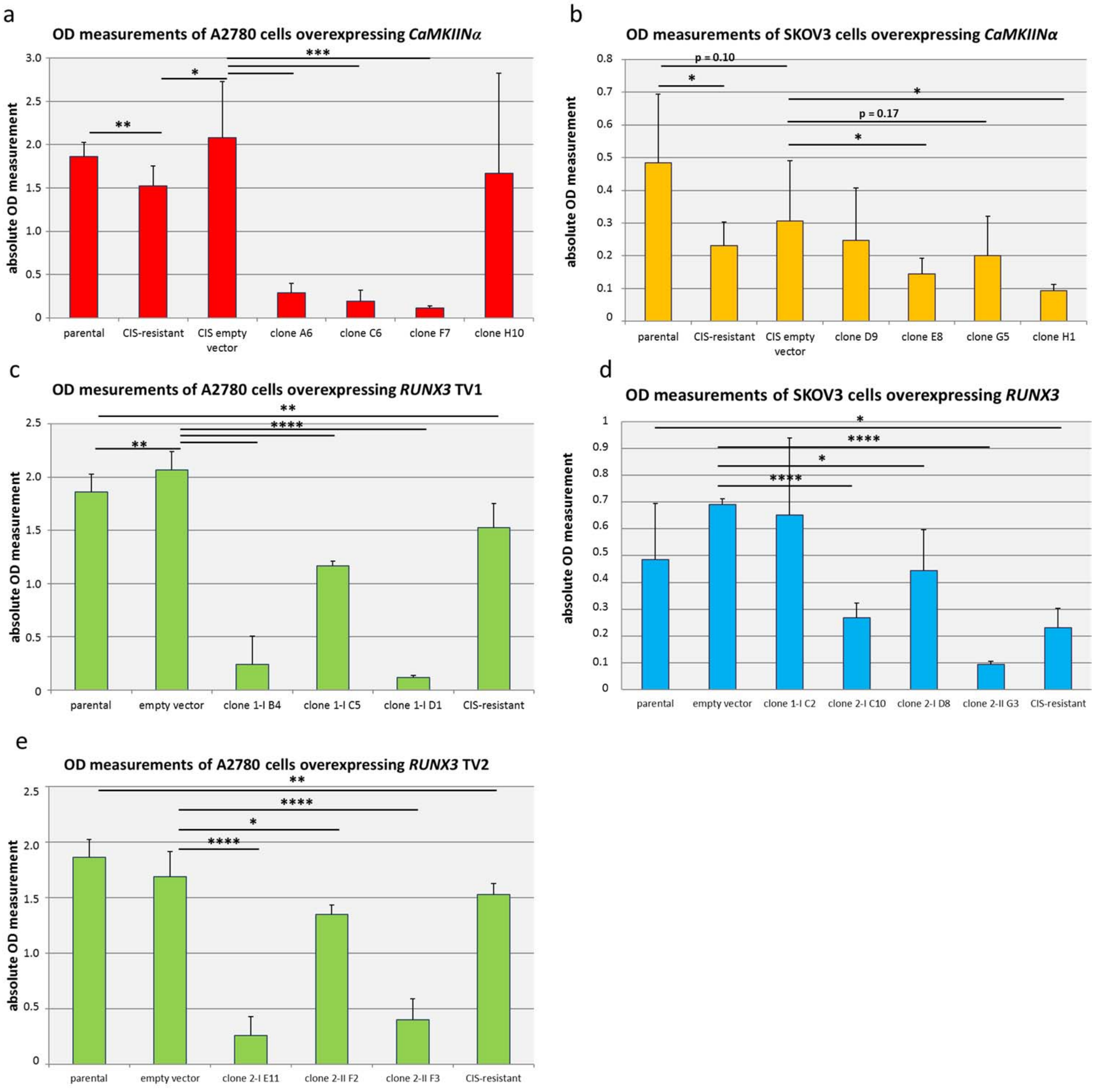

Supplementary Figure S4. Raw data of the colony-formation assay using controls and cells under *CaMKIINα* and *RUNX3* expression. Graphs showing the data of all tested *CaMKIINα* single cell clones of A2780 (a) and SKOV3 cells (b) compared to parental, CIS-resistant and empty vector control cells. An overall decrease in the ability to form colonies was observed. The absolute OD measurements of A2780 cells expressing *RUNX3* TV1 (c) and *RUNX3* TV2 (e) reflect an inhibitory effect of both *RUNX3* transcript variants on the colony forming ability in comparison to the control cells. While in SKOV3 cells the expression of TV1 led to no change, a decline of the colony number was measured when TV2 was overexpressed (d).

Figure S5

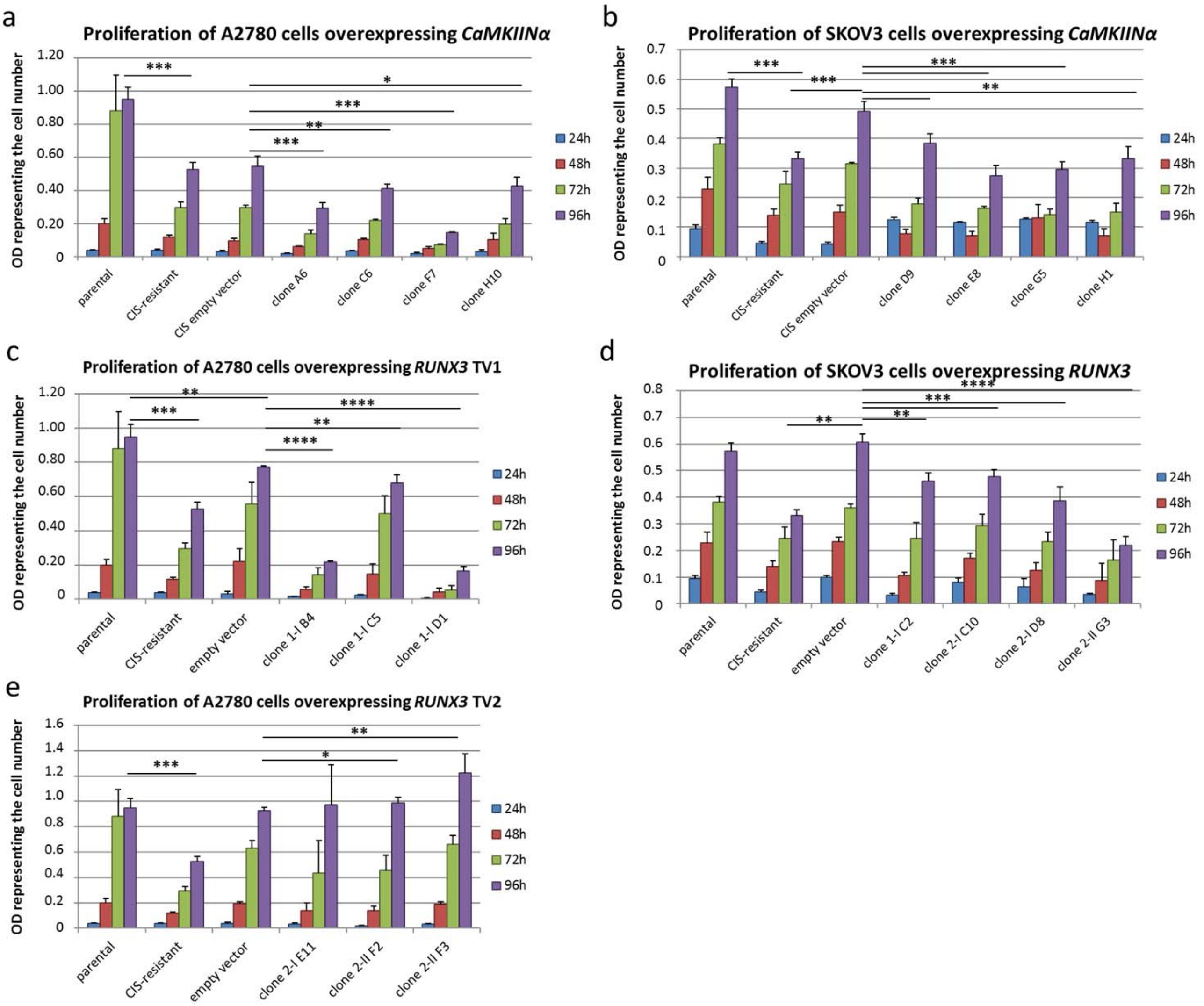

Supplementary Figure S5. Data of the MTT assay normalized to values at 2 h reflect the number of cells under *CaMKIINα* and *RUNX3* expression at 4 distinct time points. (a, b) Graphs showing the data of all tested *CaMKIINα* single cell clones of A2780 (a) and SKOV3 cells (b) compared to parental, CIS-resistant and empty vector control cells. Overall a decrease in the proliferation rate was observed. The absolute OD measurements of A2780 cells (c) and SKOV3 cells (d) expressing *RUNX3* TV1 revealed a lower proliferation in comparison to the empty vector control cells. The overexpression of *RUNX3* TV2 resulted just in A2780 cells (e; 2/3 clones)) but not in SKOV3 cells (d) to a slight increase in the proliferation of tested single cell clones.
